# PRA Melting-ICE Project: Svalbard 2022 Expeditions Report

**DOI:** 10.12688/openreseurope.17772.1

**Published:** 2024-12-04

**Authors:** Francesco Montemagno, Martina Cascone, Carlo Cardellini, Jacopo Pasotti, Elena Manini, Elisa Baldrighi, Enrico Maiero, Delia Segato, Riccardo Cerrato, Mauro Mazzola, Massimiliano Vardè, Angelina Cordone, Stefano Caliro, Iain Rudnik, Margaret Cramm, James Bradley, Donato Giovannelli

**Affiliations:** 1 Department of Biology, University of Naples Federico II, Naples, Campania, Italy; 2 Department of Physic and Geology, University of Perugia, Perugia, Italy; 3 Italian National Institute of Geophysics and Volcanology, INGV, section of Bologna, Bologna, Italy; 4 Institute for Marine Biological Resources and Biotechnologies, Italian National Research Council, CNR-IRBIM, Ancona, Italy; 5 Department of Environmental Sciences, Informatics and Statistics, Ca' Foscari University of Venice, Venice, Italy; 6 National Institute of Oceanography and Applied Geophysics, OGS, Trieste, Italy; 7 European Commission, Joint Research Centre, JRC, Ispra, Italy; 8 Institute of Polar Sciences, Italian National Research Council, CNR-ISP, Venice, Italy; 9 Institute of Atmospheric Pollution Research, Italian National Research Council, CNR-IIA, Firenze, Italy; 10 Department of Earth Science, University of Pisa, Pisa, Italy; 11 Institute of Polar Sciences, Italian National Research Council, CNR-ISP, Bologna, Italy; 12 Italian National Institute of Geophysics and Volcanology, INGV, Naples, Italy; 13 British Antarctic Survey, High Cross, Madingley Road, Cambridge, UK; 14 School of Geography, Queen Mary University of London, Mile End Road, London, UK; 15 Aix Marseille Univ, Université de Toulon, CNRS, IRD, MIO, Marseille, France; 16 School of Biological and Behavioural Sciences, Queen Mary University of London, London, England, UK; 17 Marine Chemistry and Geochemistry Department, Woods Hole Oceanographic Institution, Woods Hole, Massachusetts, USA; 18 Earth-Life Science Institute, ELSI, Tokyo Institute of Technology, Tokyo, Japan; 19 Department of Marine and Coastal Science, Rutgers University New Brunswick, New Brunswick, New Jersey, USA

**Keywords:** Expedition report, Permafrost, Arctic, Ny-Ålesund, Climate change, Microbiology, Greenhouse gasses

## Abstract

Arctic regions are among the fastest warming areas of the planet. Increasing average temperatures over the last five decades have deepened the thawing of the upper-most layer of permafrost across the Arctic, which contains significant amounts of organic carbon. The progressive deepening of seasonal thawing releases carbon that is used by active microorganisms which also produce greenhouse gases, potentially onsetting a positive feedback on global warming. Despite their importance in controlling organic matter degradation and greenhouse gas fluxes to the atmosphere, there is a lack of data on activity and dynamics of microbial communities in High Arctic soils in response to seasonal thaw. This report describes three specific expeditions performed on the Svalbard archipelago, carried out within the framework of the PRA (Italian Arctic Research Program) project Melting-ICE, performed between February and October 2022, reporting site characteristics and samples collected. The project aims to investigate the diversity and activity of active layer microbial communities across a full season thaw cycle, correlating microbial diversity with gas fluxes and composition. During these expeditions, a total of eight different sites were selected to investigate the microbiology and geochemistry of soils, as well as to estimate the gas fluxes from the soil to the atmosphere. The data collected in the field, combined with the results obtained in the laboratory, will provide a snapshot of the seasonal activity of the microbial communities present in the permafrost’s active layer. The three campaigns will provide data to estimate the impact of permafrost melting on the carbon cycle and the role of microorganisms in the release of greenhouse gases.

## Plain language summary

The "Melting-ICE" project examines how the Arctic, particularly the Svalbard archipelago, is responding to rapid climate changes. Located in the Arctic Ocean between latitudes 74°N and 81°N, this group of islands is undergoing faster temperature rises and glacier retreats than many other parts of the world. Approximately 60% of the archipelago is covered with glaciers, and the non-glaciated areas contain permafrost a permanently frozen ground that can be extremely deep. As the region warms, this permafrost is thawing, releasing carbon and greenhouse gases (such as CO _2_ and methane) that had been trapped in the ice for thousands of years. These gases contribute to the greenhouse effect, further accelerating global warming. A key role in this process is played by microbial communities in the soil, which consume the released carbon and produce greenhouse gases. The project conducted three scientific expeditions to the Ny-Ålesund area in Svalbard from winter to autumn 2022 to study how microbial activity varies with the seasons and its impacts on greenhouse gas emissions. Through field experiments and laboratory analysis, researchers gathered data to better understand how changes in permafrost affect the carbon cycle and greenhouse gas release. These studies are crucial for predicting and managing the effects of climate changes in the Arctic and globally.

## Introduction

The Arctic is warming faster than any other region on the planet ( Graversen *et al.*, 2008) and has experienced extreme positive temperature anomalies constantly increasing in the last three decades ( Graversen *et al.*, 2008; Walsh, 2014). As a result, the Arctic has lost significant amounts of summer ice cover and permafrost ( Graversen *et al.*, 2008; Serreze & Barry, 2011; Tepes *et al.*, 2021). In the last few decades, permafrost temperatures have risen across the Arctic ( Smith *et al.*, 2022), and the Global Terrestrial Observing System (GTOS) has identified permafrost as one of six cryospheric indicators of global climate change ( Streletskiy *et al.*, 2017). Permafrost thaw has liberated organic carbon, nutrient and trace elements that have been trapped for hundreds to many thousands of years in a frozen state, stimulating microbial activity and increasing the flux of CO _2_, N _2_O, and CH _4_ to the atmosphere ( Hayes *et al.*, 2014). While these processes have been extensively studied in soils during the summer months (e.g. Jansson & Taş, 2014; Sipes *et al.*, 2022), the activity of microbial communities and the associated greenhouse gas fluxes during the winter have received comparatively little attention ( Graham *et al.*, 2012; Nikrad *et al.*, 2016; Schostag *et al.*, 2015; Vigneron *et al.*, 2019), and data on full seasonal patterns of microbial activity and carbon cycling are scarce. Additionally, the direct coupling between carbon and nutrient utilization by soil microbial communities with the exports to the marine ecosystems, and how these can significantly alter the biogeochemical balance of the area, are significantly less understood ( Walvoord & Striegl, 2007).

The response of the Arctic ecosystems to increased melting events is mediated by the microbial community response to these events ( Graham *et al.*, 2012). Processes such as respiration, fermentation, methanogenesis, and methane oxidation, together with the efficiency of carbon transfer to higher trophic levels, significantly influence the ultimate fate of the newly released carbon and nutrients ( Jansson & Taş, 2014). Characterizing the microbial communities and soil gas fluxes across seasons is of paramount importance to better constrain the response of Arctic soils to ongoing climate change ( Graham *et al.*, 2012). While the quality and quantity of carbon released during the summer (and winter) melting events is a strong control on microbial activity ( Miner *et al.*, 2022), the release of nutrients and trace elements might place additional constraints on the microbial community response, potentially influencing the type of metabolic processes that are active ( Giovannelli, 2023).

The Svalbard archipelago, located in the Arctic Ocean between 74 °N and 81 °N, is highly glaciated and it is experiencing rapid increases in temperature and glacial retreat, making it a model system for future changes to the rest of the Arctic ( Martín-Moreno *et al.*, 2017). Approximately 60 % of the archipelago surface is covered by glaciers, leaving a periglacial area where about 25,000 km ^2^ is covered by permafrost making Svalbard the largest permafrost area in Europe. The archipelago has a continuous permafrost layer that reaches up to 100 m depth in coastal areas and greater than 500 m in mountainous areas. Svalbard permafrost, also known as “warm permafrost”, is particularly sensitive to climate change, since its temperature is close to 0 °C ( Humlum *et al.*, 2003; Isaksen *et al.*, 2007; Keating *et al.*, 2018). Mountain topography also has a role in making Svalbard permafrost more sensitive to climate change, as variables like altitude, slope, and snow cover can cause changes in the ground temperature and affect the permafrost thickness. Temperatures in the area have steadily increased to about ~3 °C above the seasonal mean, and winter snow cover has been rapidly declining ( Isaksen *et al.*, 2007; Vickers *et al.*, 2021). The increasing temperatures have resulted in longer and deeper permafrost thawing events, increasing greenhouse gas emissions in the area. While extensively studied during the summer months, a handful of studies have investigated the soil microbial communities during the winter months in Svalbard ( Loganathachetti *et al.*, 2022; Schostag *et al.*, 2015).

The project Melting-ICE was designed to provide an integrated view of the active layers of microbial communities during the winter, summer and autumn seasons while spanning the land-to-sea transition. The project will generate data on the microbial diversity in the active layer, together with quantitative estimates of greenhouse gas fluxes and isotopic signatures. Three independent field expeditions have been conducted to compare the fluxes and microbial diversity during the Arctic winter with the conditions present at the beginning of the summer melt season and the beginning of Autumn freeze events. This report describes the three expeditions to the Ny-Ålesund area, on the island of Spitsbergen, Svalbard archipelago, Norway.

## The expedition team and logistics

The project team is composed of a team of interdisciplinary scientists spanning the microbiology, biogeochemistry, microbial ecology and gas geochemistry fields. The expedition was hosted by the Italian “Dirigibile Italia” Arctic Station managed by the Institute of Polar Sciences of the Italian National Research Council and the UK Arctic Research Station, both located in Ny-Ålesund, which provided logistic support. The field science party was responsible for the sample collection and the field measurements. Three sampling expeditions were performed, in February 2022, June 2022 and September 2022, coinciding with a full active layer thaw cycle.

During the winter expedition (i.e., February 2022), given the extremely low temperatures, drilling operations and gas flux measurements were particularly challenging. The Field Science Party performed sampling operations for 17 days with windchill temperatures down to -38 °C (air temperature of -28 °C). Sampling operations were suspended for two days during bad weather conditions when windchill temperatures reached as low as -44 °C. Recovery of intact core samples was difficult due to the fast freezing of the core within the core drill barrel during operation, and only a few intact cores were recovered. For the rest of the locations, coring was performed in sections and the recovery was limited to loose material dislodged from the core barrel. Gas flux measurements were limited by battery life at low temperatures, in spite of the isolation and heat sources placed in proximity of the instruments. The Onshore Science Party was responsible for supporting the logistics and preparation of the expedition as well as collaborating on sample processing and analysis.

**Figure 1.**
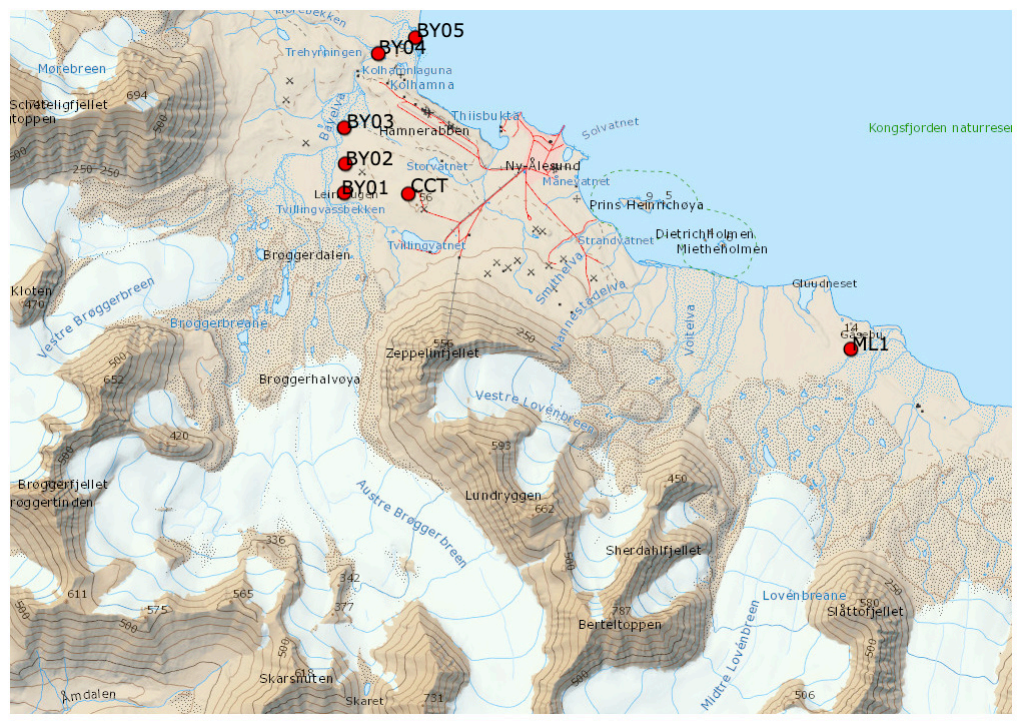
Map of the sampled area with the location of the sampling sites (red dots) and the position of the Ny-Ålesund International Research Station ( https://nyalesundresearch.no/). The topographic map was obtained from the Norwegian Polar Institute ( https://toposvalbard.npolar.no/).

The second 10 days expedition, performed in June, allowed the collection of sediment and fluid samples from the 6 sites originally chosen during the first campaign, and from an additional 7 ^th^ site. Air temperature ranged between 5 and 8 °C and snow cover was absent. Soils were soaked with meltwater. The 7 days of fieldwork originally planned were reduced to 6 due to the presence of several polar bears nearby the BY4 and BY5 sites ( Figure 1).

The fall sampling expedition was carried out at the end of September. Air temperature ranged between 2 and 5 °C, and snow cover was absent. Soil was mostly dry. During this third field expedition, all 7 sites were sampled. ( Table 8)

*Field Science Party composition:* Francesco Montemagno, Martina Cascone, Carlo Cardellini (CoI of the project), James Bradley, Margaret Cramm, Jacopo Pasotti, Enrico Maiero, Delia Segato, Riccardo Cerrato (Italian Acting Station Leader), Massimiliano Vardè, Ian Rudkin, Donato Giovannelli (Expedition Leader and PI of the project).

*Support Science Party composition:* Elena Manini (CoI of the project), Elisa Baldrighi, Mauro Mazzola, Angelina Cordone (CoI of the project), Stefano Caliro.

## Geological and environmental settings

**Figure 2.**
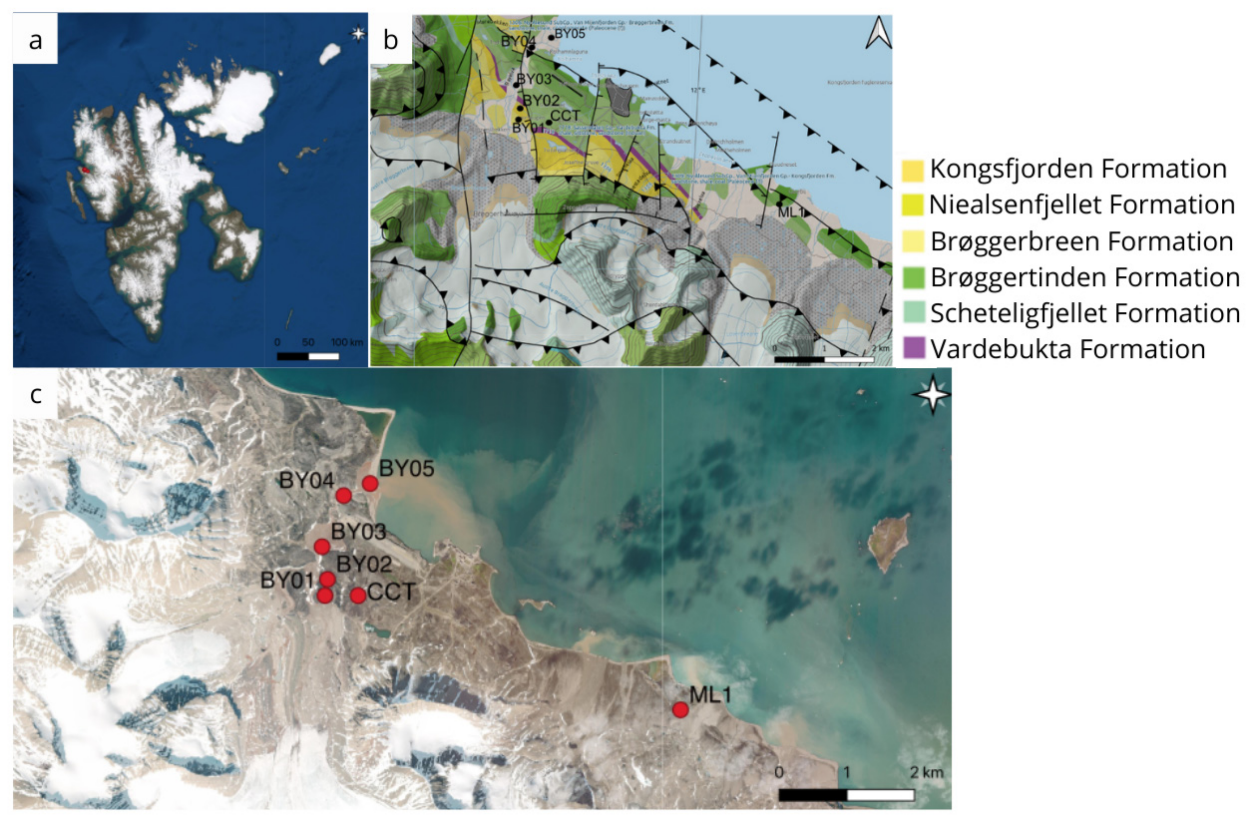
( **a**) Satellite image of Svalbard archipelago, where the sampling took place ( **b**) Geological context of the sampling area around Ny-Ålesund, Svalbard. The map was obtained from the site Svalbard Kartet ( https://geokart.npolar.no/Html5Viewer/index.html?viewer=Svalbardkartet) ( **c**) Satellite image of the Ny-Ålesund area. The sampling sites are reported with the red dots. This panel was created using Google Maps for image ( **a**) and ( **c**), and QGIS ( https://www.qgis.org/en/site/index.html) for image ( **b**).

The sampling was performed in the Ny-Ålesund (Svalbard) area ( Figure 1) that is located on the Brøggeralvøya on the SW shore of Kongsfjorden, in the northwestern part of Spitsbergen, Svalbard. The area sits on a plateau, surrounded by three main mountains, Zeppelinfjellet (556 m a.s.l.), Berteltoppen (785 m a.s.l.), and Scheteligfjellet (719 m a.s.l.) and by the glacial valleys of Austre-Vestre Brøggerbreen, Vestre Lovénbreen and Midtre Lovénbreen. The Paleocene sandstone, shales, conglomerates, and coal-rich sequence is overlaid by sedimentary rocks from the Middle Carboniferous-Early Triassic ( Figure 2) ( Van Everdingen, 1981). The bedrock sequences that include conglomerate, sandstone, dolomite, shale, chert, and limestone are involved in a NE-verging thrust fault and NNE-SSW high-angle faults, with closely spaced joints and fractures systems ( Cianfarra & Salvini, 2016). The area is also interested by conspicuous moraine deposits and glacier forefield deposits, as Ny-Ålesund is surrounded by a large number of large ice streams, Kongsvegen, Kronebreen, Kongsbreen and Conwaybreen, and glaciers, like Austre Brøggerbreen, Vestre Brøggerbreen, Lovénbreen, Pedersbreen, Botnbreen and Midtre Lovénbreen. The Austre-Vestre Brøggerbreen and Vestre Lovénbreen glaciers melt waters converge in the Bayelva river, discharging directly in the Kingsfjord just NW of Ny-Ålesund.

The Bayelva river is collated in two major glacier valleys that result from the recession of the Brøggerbreen during the present interglacial period ( Sadiq *et al.*, 2022). The sampling sites were either placed within moraine deposits or meltwater sedimentary deposits. A sixth site was sampled south-east of Ny-Ålesund, in the forefield of the polythermal valley glacier Midtre Lovénbreen, in an area more representative of the arctic tundra and external to the moraine fields, and close to a temporary year-round monitoring observatory ( Cimpoiasu *et al.*, 2024a; Cimpoiasu *et al.*, 2024b). A permanent permafrost monitoring site has been active in proximity of site BY01 since 1998, reporting a yearly average temperature of -2.8 °C ( Boike *et al.*, 2018). The area is also monitored by the Italian eddy-covariance system hosted at the “Amundsen-Nobile Climate Change Tower” located eastward of BY01 and in close proximity to site CCT ( Figure 1). The average temperature in the region in January ranges between -17.0 and -3.8 °C, while the mean July temperature ranges from 4.6 to 6.9 °C ( Maturilli *et al.*, 2012).

## Sampling methods

### Sediment collection and handling

The sampling was carried out along a transect that flanks the Bayelva river, with the exception of a site located in the forefield of the Midtre Lovénbreen glacier. ( Table 1) The exact location of each site was decided in the field during the winter, and at times several attempts were made digging snow pits with the aim of finding areas of soil with minimal rock cover to allow for easy drilling of the permafrost. At each location a snowpit of about 2×3 m was dug to allow at least two people to work inside. The thickness of the snow removed to create the snowpit was recorded, and varied between 19 and 105 cm per site, although in some attempts up to 185 cm of snow were removed. Permafrost depth was also recorded at each site ( Table 2).

**Table 1.** Site coordinates and soil temperatures.

				Soil temperature			
SiteID	Site name	Latitude (°N)	Longitude (°E)	Expedition 1 @ 2 cm (°C)	Expedition 2 @ 2 cm (°C)	Expedition 3 @ 2 cm (°C)	Notes
BY1	Bayelva 1	78.92102	11.83289	-1.6	7.8	4.3	Bayelva permafrost observatory ( Boike *et al*., 2018)
BY2	Bayelva 2	78.92315	11.83403	-3.4	5.8	4.9	
BY3	Bayelva 3	78.92743	11.82908	-5.4	7.7	4.9	
BY3A	Bayelva 3	78.92683	11.82886	NA	10.7	5.2	
BY4	Bayelva 4	78.93416	11.84456	-2.8	9.2	2.7	
BY5	Bayelva 5	78.93651	11.85860	-6.5	7.7	2.2	
ML1	Midtre Lovénbreen 1	78.90833	12.08157	-2.4	8	3.6	ST. 3 form J Bradley (QMUL, UK)
CCT	Climate ChangeTower	78.921367	11.865867	NA	8.1	5	

**Table 2.** Average depths of the active layer in the three expeditions for each site.

		Active layer depth (cm)		
SiteID	Site name	Expedition 1	Expedition 2	Expedition 3
BY1	Bayelva 1	0	30	35
BY2	Bayelva 2	0	20	26
BY3	Bayelva 3	0	5	20
BY3A	Bayelva 3	NA	20	23
BY4	Bayelva 4	0	25	27
BY5	Bayelva 5	0	15	31
ML1	Midtre Lovénbreen 1	0	35	33
CCT	Climate ChangeTower	NA	40	40

**Figure 3.**
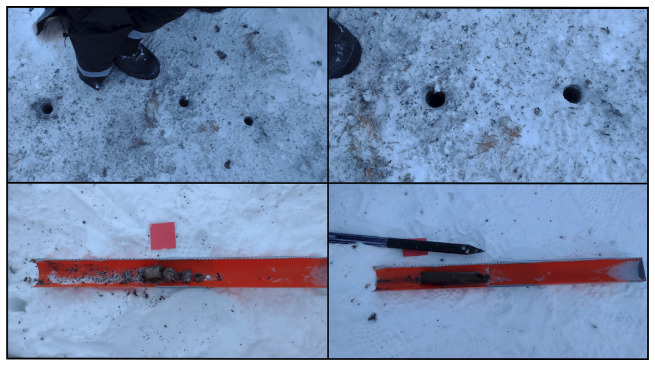
Panel showing snow pit and core operations. Photograph taken by author FM for this publication.

**Figure 4.**
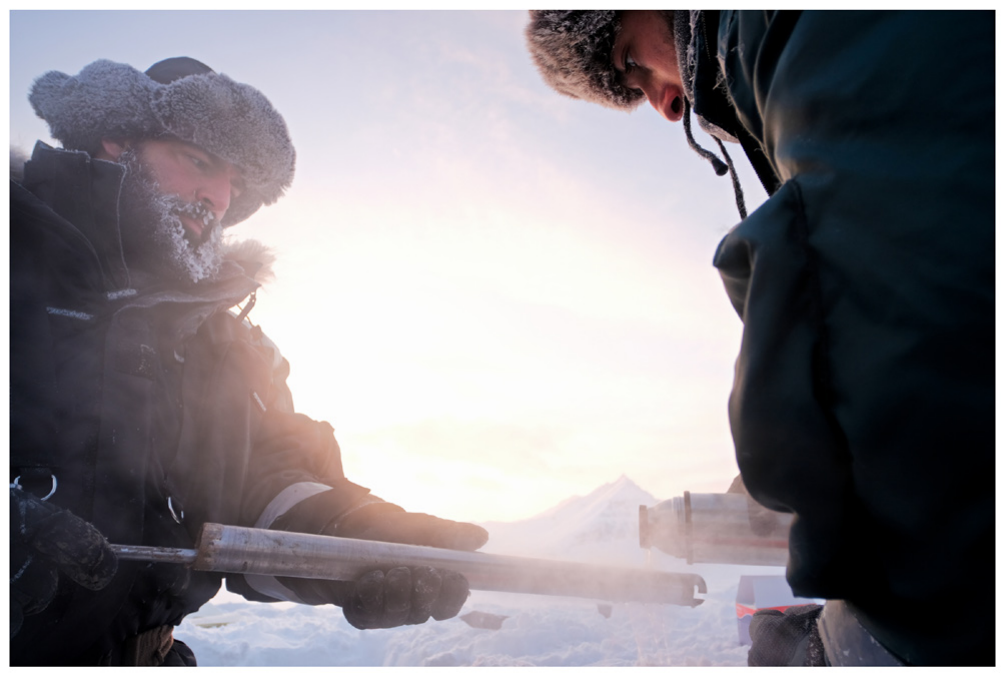
Given the low air temperatures, the cores were freezing inside the barrel expanding and hindering easy recovery. Hot water was used to warm the external surface of the core barrel to allow for core extrusion. Photograph taken by a member of the first expedition JP for this publication.

Coring of the permafrost was performed using a portable driller (Shaw Drill Sondaj, https://backpackdrill.com) equipped with a 41 mm diameter core barrel and a diamond drill bit. At each site three independent boreholes were drilled ( Figure 3, Table 3). The presence of rocks at depth prevented complete recovery of all the cores. Once the core was extracted from the ground, the low external temperature immediately froze the material, often preventing the recovery of the intact core. Hot water was used to warm the external surface of the core barrel to allow core recovery ( Figure 4). The cores were extracted by placing it on a sterile housing for photographic documentation and length measurements and then transferred into sterile Whirl-Pak sample bags. Either continuous intact cores or multiple core sections were recovered from each borehole. Cores were preserved frozen at -20 °C and returned to the laboratory for further analysis.

**Table 3.** List of boreholes and recovered cores in the first expedition, February 2022.

SiteID	Site name	CollectionID	Borehole	Drill depth (cm)	Core Recovery (%)
BY1	Bayelva 1	BY1220225	a	0–19	79
BY1	Bayelva 1	BY1220225	b	0–12	20
BY1	Bayelva 1	BY1220225	b	12–20	50
BY1	Bayelva 1	BY1220225	c	0–19	68
BY2	Bayelva 2	BY2220227	a	0–14	71
BY2	Bayelva 2	BY2220227	a	14–25	145 *
BY2	Bayelva 2	BY2220227	b	0–14	121 *
BY2	Bayelva 2	BY2220227	c	0–17.5	91
BY3	Bayelva 3	BY3220227	a	0–14	46
BY3	Bayelva 3	BY3220227	a	14–19	140 *
BY3	Bayelva 3	BY3220227	b	0–13	NA
BY3	Bayelva 3	BY3220227	c	0–24	NA
BY3	Bayelva 3	BY3220227	c	24–32	175 **
BY4	Bayelva 4	BY4220228	a	0–15	87
BY4	Bayelva 4	BY4220228	a	15–21	83
BY4	Bayelva 4	BY4220228	b	0–11	54
BY4	Bayelva 4	BY4220228	c	0–15	57
BY5	Bayelva 5	BY5220228	a	0–10	NA
BY5	Bayelva 5	BY5220228	a	10–23.5	111 **
ML1	Midtre Lovénbreen 1	ML122031	a	0–17.5	NA
ML1	Midtre Lovénbreen 1	ML122031	a	17.5–22	NA
ML1	Midtre Lovénbreen 1	ML122031	a	22–27	130 **
ML1	Midtre Lovénbreen 1	ML122031	b	0–11	NA
ML1	Midtre Lovénbreen 1	ML122031	b	11–18	157 **
ML1	Midtre Lovénbreen 1	ML122031	c	0–7.3	NA

*Core expansion likely due to freezing upon retrieval and presence of unconsolidated layers **Core recovery is overestimated due to collection of fragments from the drilling of the above section

**Figure 5.**
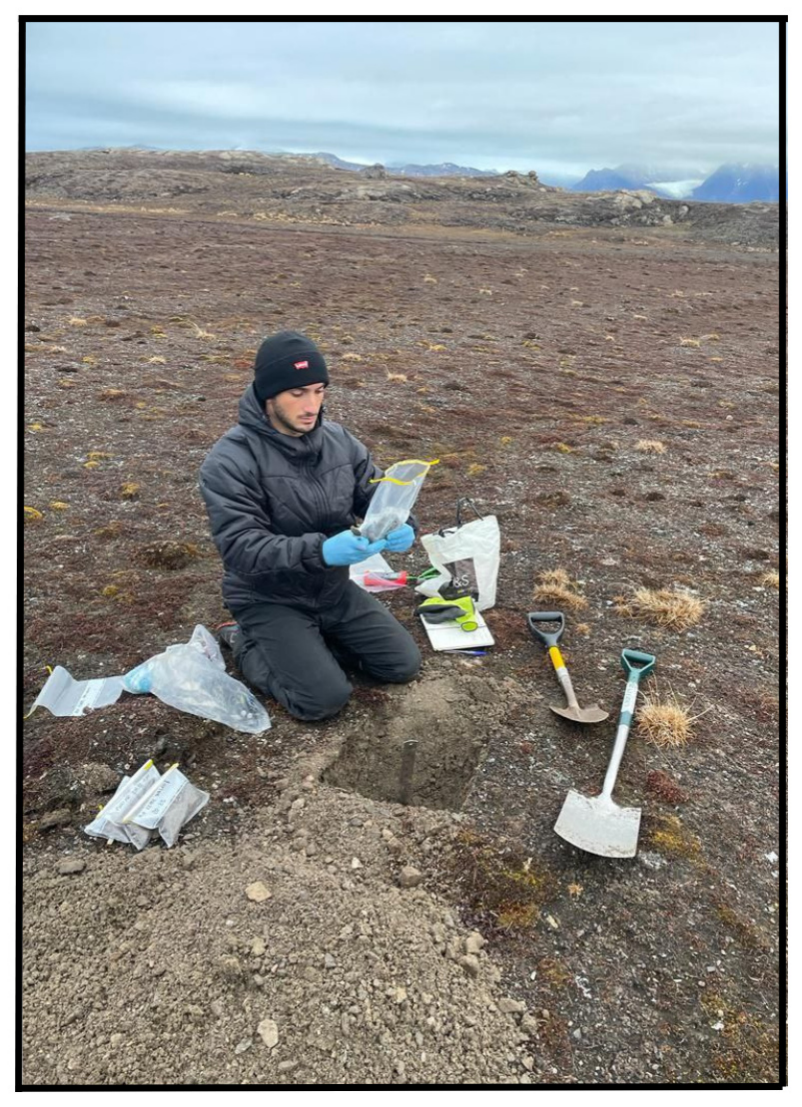
During the second and third expedition, the soil was collected and placed into a Whirl-Pak bag. Photograph taken by a member of third expedition MC for this publication.

During the summer, we revisited the sampling sites previously surveyed in winter, and added one additional location ( Table 1). Utilizing shovels and hand tools, we excavated rectangular soil pits, consistently digging from one side to maintain an intact wall. This approach aimed to facilitate soil sampling without the risk of disturbance or mixing. The excavated pits, ranging from 20 to 40 cm in depth, provided a stratified profile for comprehensive analysis ( Table 4 and Table 5). At various depths within the excavated pits, sediment samples were collected and subsequently preserved in Whirl-Pak bags ( Figure 5). All sampled soils are classified as Gelisoil ( IUSS Working Group WRB, 2022).

**Table 4.** List of sites and sampled depths in the second expedition, June 2022.

SiteID	Site name	CollectionID	Depths sampled (cm)
BY1	Bayelva 1	BY1220614	0–5 / 10–15 / 20–30
BY2	Bayelva 2	BY2220614	0–5 / 10–15 / 20–25
BY3	Bayelva 3	BY3220614	0–5
BY3A	Bayelva 3	BY3A220614	0–5 / 10–15 / 20
BY4	Bayelva 4	BY4220619	0–5 / 10–15 / 20–25
BY5	Bayelva 5	BY5220619	0–5 / 10–15
ML1	Midtre Lovénbreen 1	ML1220615	0–5 / 10–15 / 20–30 / 30–40
CCT	Climate Change Tower	CCT220618	0–5 / 10–15 / 20–30 / 30–35

**Table 5.** List of sites and sampled depths in the third expedition, September 2022.

SiteID	Site name	CollectionID	Depths sampled (cm)
BY1	Bayelva 1	BY1220930	0–5 / 10–15 / 20–25 / 30–35
BY2	Bayelva 2	BY220930	0–5 / 10–15 / 20–26
BY3	Bayelva 3	BY3220930	0–5 / 10–15 / 20
BY3A	Bayelva 3	BY3A220930	0–5 / 10–15 / 20–23
BY4	Bayelva 4	BY422103	0–5 / 10–15 / 20–27
BY5	Bayelva 5	BY422103	0–5 / 10–15 / 20–25 / 30–31
ML1	Midtre Lovénbreen 1	ML122101	0–5 / 10–15 / 20–25 / 30–35 / 40
CCT	Climate Change Tower	CCT220930	0–5 / 10–15 / 20–25 / 30–33

### Gas flux measurements

The diffuse gas flux from the soil surface, and at some sites from the snow surface, was measured using the accumulation chamber method ( Chiodini *et al.*, 1998). Three different instrumentations were used. For the first expedition: 1) an in house built instrument by the University of Perugia, equipped with a LI-COR LI-820 Infra-Red detector (IR) for CO _2_ set in a range from 0 to 5,000 ppm, connected with an accumulation chamber of ~2.8 L volume; 2) a West System ^©^ instrument, equipped with a LI-COR 820 IR for CO _2_ set in a range from 0 to 20,000 ppm, with a Tunable Diode Laser Absorption Spectroscopy (TDLAS) detector for CH _4_ and an electrochemical sensor for H _2_S, connected with an accumulation chamber of ~2.8 L volume. For the second and the third expedition: 1) an in house built instrument by the University of Perugia, equipped with a LI-COR LI-820 Infra-Red detector (IR) for CO _2_ set in a range from 0 to 5,000 ppm, connected with an accumulation chamber of ~2.8 L volume; 2) a Thearen © instrument, equipped with a LI-COR 830 IR for CO _2_ set in a range from 0 to 20,000 ppm and a Tunable Diode Laser Absorption Spectroscopy (TDLAS) detector for CH _4 _connected with an accumulation chamber of ~3.6 L volume. For the first survey the gas flux measurements from the permafrost surface were carried out after removing the layer of snow and ice present above the permafrost, which was instead left undisturbed. Measurements were performed at the sites of permafrost coring and nearby the Italian Arctic station “Dirigibile Italia”, for comparison and instrumentation testing.

### Gas samples collection

**Figure 6.**
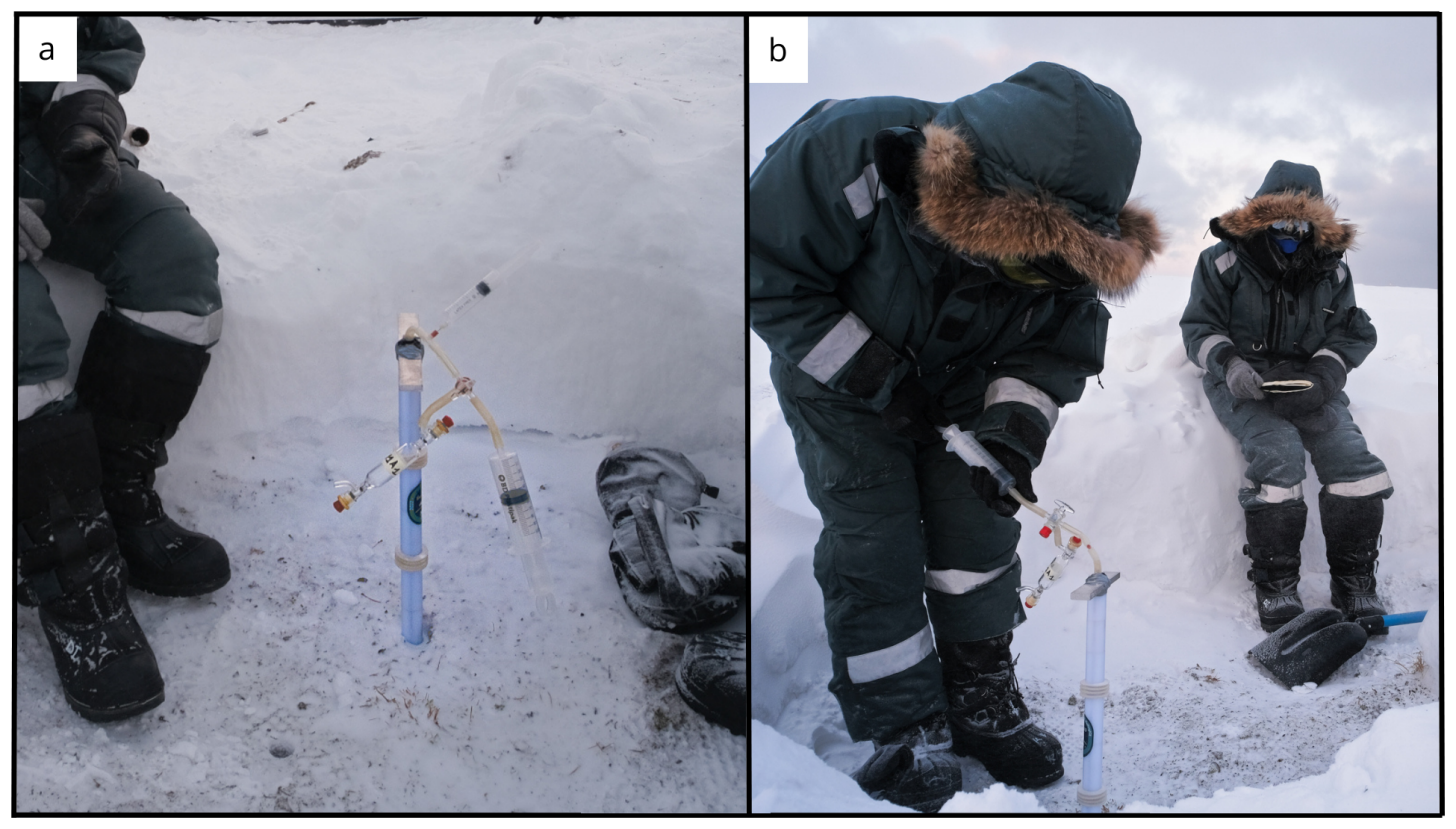
Permafrost borehole gas sampling apparatus. ( **a**) Detailed view of gas sampling probe designed for the project. Inside the plastic body is placed a 1/8 inch stainless steel capillary. ( **b**) the sampling probe inserted into a borehole during gas sampling operations. Photographs taken by a member of the first expedition JP for this publication.

In the first expedition interstitial gas was sampled from the boreholes using a dedicated sampling probe ( Figure 6a). The probe is made by an external body of plastic materials containing a stainless-steel capillary of about 3 mm of diameter. The probe is equipped with a series of 4 silicone gaskets at 2 cm from the bottom and every 15 cm, to allow the fitting of the probe to the drilled hole and preventing/limiting air contamination. The gas was collected in both 20 mL glass vials and pre-evacuated vacutainer for laboratory analyses of its chemical composition and of the isotopic composition of the carbon of CO _2_. The gas was collected after purging the line using a syringe connected to a 3-way valve ( Figure 6b). In the other two expeditions where coring was not performed, a stainless steel probe of 5 mm of diameter was used to reach the desired depth in the soil and, in addition to vacutainer and glass vials, Tedlar and multi-layer foil gas sampling bags of 1 L were used to collect soil gas ( Table 6, Table 7 and Table 8).

**Table 6.** List of collected gas samples for the first expedition, February 2022.

SiteID	Site name	CollectionID	Borehole	Depth (cm)	AC sample	Glass vial	Vacutainer	Notes
BY1	Bayelva 1	F1A			x		x	
BY1	Bayelva 1	F1B			x		x	
BY1	Bayelva 1	BY1A	a	19		x		
BY1	Bayelva 1	BY1B	a	19		x		
BY1	Bayelva 1	BY1A	a	19			x	
BY1	Bayelva 1	BY1B	a	19			x	
BY2	Bayelva 2	BY2A1	a	25			x	
BY2	Bayelva 2	BY2A1	a	25		x		
BY2	Bayelva 2	BY2A2	b	14			x	
BY2	Bayelva 2	BY2A2	b	14		x		
BY2	Bayelva 2	BY2F10			x			
BY2	Bayelva 2	BY2F11			x			
BY2	Bayelva 2	BY2F12			x			bad sample
BY2	Bayelva 2	BY2F13			x			
BY2	Bayelva 2	BY2F14			x			
BY3	Bayelva 3	BY3A1					x	
BY3	Bayelva 3	BY3A1				x		
BY3	Bayelva 3	BY3F10	a	19	x			
BY3	Bayelva 3	BY3F11	a	19	x			
BY4	Bayelva 4	BY4A1	a	21		x		
BY4	Bayelva 4	BY4A2	a	21		x		
BY4	Bayelva 4	BY4A1	a	21			x	
BY4	Bayelva 4	BY4A1-B	a	21			x	
BY4	Bayelva 4	BY4F1			x			
BY4	Bayelva 4	BY4F4			x			
BY5	Bayelva 5	BY5A1	a	23.5			x	
BY5	Bayelva 5	BY5A1-B	a	23.5			x	
BY5	Bayelva 5	BY5A2	a	23.5			x	
BY5	Bayelva 5	BY5A1	a	23.5		x		
BY5	Bayelva 5	BY5A1-B	a	23.5		x		
BY5	Bayelva 5	BY5F1			x		x	On the snow
BY5	Bayelva 5	BY5F3			x		x	On the snow
BY5	Bayelva 5	BY5F4			x		x	On the snow
BY5	Bayelva 5	BY5F10			x		x	
BY5	Bayelva 5	BY5F11			x		x	
ML1	Midtre Lovénbreen 1	J3F1			x		x	No Flux measure
ML1	Midtre Lovénbreen 1	J3F2			x		x	No Flux measure
ML1	Midtre Lovénbreen 1	J3F3			x		x	No Flux measure
ML1	Midtre Lovénbreen 1	J3F4			x		x	No Flux measure
ML1	Midtre Lovénbreen 1	J3F6			x		x	No Flux measure
ML1	Midtre Lovénbreen 1	J3F7			x		x	No Flux measure
ML1	Midtre Lovénbreen 1	J3F8			x		x	No Flux measure
ML1	Midtre Lovénbreen 1	ML1A-1	a	17.5		x		
ML1	Midtre Lovénbreen 1	ML1A-1A	a	17.5			x	
ML1	Midtre Lovénbreen 1	ML1A-1B	a	17.5			x	
ML1	Midtre Lovénbreen 1	ML1A-2	a	22.0		x		
ML1	Midtre Lovénbreen 1	ML1A-2A	a	22			x	
ML1	Midtre Lovénbreen 1	ML1A-3A	a	27		x		
ML1	Midtre Lovénbreen 1	ML1A-3B	a	27		x		
ML1	Midtre Lovénbreen 1	ML1A-3A	a	27			x	
ML1	Midtre Lovénbreen 1	ML1A-3B	a	27			x	
ML1	Midtre Lovénbreen 1	ML1F10			x		x	
ML1	Midtre Lovénbreen 1	ML1F11			x		x	
ML1	Midtre Lovénbreen 1	ML1F12			x		x	
ML1	Midtre Lovénbreen 1	ML1F13			x		x	
BASE	Base 3	BASEF1			x			Ny-Ålesund base
BASE	Base 3	BASEF2			x			Ny-Ålesund base

**Table 7.** List of collected gas samples for the second expedition, June 2022.

SiteID	Site name	CollectionID	Depth (cm)	AC sample	Glass vial	Vacutainer	Gas bag	Notes
BY1		BY1B	30		x	x		water @40 cm
BY1	Bayelva 1	BY1B1	30			x		water @40 cm
BY1	Bayelva 1	BY1B2	30			x		water @40 cm
BY1	Bayelva 1	BY1A1	20				x	water @40 cm
BY1	Bayelva 1	BY1B	30				x	water @40 cm
BY1	Bayelva 1	BY1F1		x		x		
BY1	Bayelva 1	BY1F3		x		x		
BY1	Bayelva 1	BY1F5		x		x		
BY1	Bayelva 1	BY1F2		x		x		
BY1	Bayelva 1	BY1F4		x		x		
BY1	Bayelva 1	BY1F6		x		x		
BY2	Bayelva 2	BY2A1	20			x		
BY2	Bayelva 2	BY2A2	20			x		
BY2	Bayelva 2	BY2	20				x	
BY3A	Bayelva 3A	BY3A1	20			x		wet soil
BY3A	Bayelva 3A	BY3A2	40			x		wet soil
BY3A	Bayelva 3A	BY3AF1		x		x		wet soil
BY3A	Bayelva 3A	BY3AF3		x		x		wet soil
BY3A	Bayelva 3A	BY3AF2		x		x		wet soil
BY3A	Bayelva 3A	BY3AF4		x		x		
BY4	Bayelva 4	BY4A1	40			x		
BY4	Bayelva 4	BY4A2	40			x		
BY4	Bayelva 4	BY4A	40		x		x	
BY4	Bayelva 4	BY4B1	20			x		
BY4	Bayelva 4	BY4B2	20			x		
BY4	Bayelva 4	BY4B3	20			x		
BY4	Bayelva 4	BY4B	20		x		x	
BY4	Bayelva 4	BY4F1		x		x		
BY4	Bayelva 4	BY4F2		x		x		
BY4	Bayelva 4	BY4F3		x		x		
BY4	Bayelva 4	BY4F4		x		x		
BY5	Bayelva 4	BY5A	40			x		
BY5	Bayelva 4	BY5B	40			x		
BY5	Bayelva 4	BY5	40		x		x	
BY5	Bayelva 4	BY5F		x				
BY5	Bayelva 4	BY5F11		x				
ML1	Midtre Lovénbreen 1	ML1A1	30			x		water @>30cm
ML1	Midtre Lovénbreen 1	ML1A2	30			x		water @>30cm
ML1	Midtre Lovénbreen 1	ML1A1	30		x		x	water @>30cm
ML1	Midtre Lovénbreen 1	ML1F1		x				
ML1	Midtre Lovénbreen 1	ML1F2		x				
ML1	Midtre Lovénbreen 1	ML1F3		x				
M1L	Midtre Lovénbreen 1	ML1F4		x				
CCT	Climate Change Tower	CCT1A1	40			x		
CCT	Climate Change Tower	CCT1A2	40			x		
CCT	Climate Change Tower	CCT1A	40		x		x	
CCT	Climate Change Tower	CCT1B1	20			x		
CCT	Climate Change Tower	CCT1B2	20			x		
CCT	Climate Change Tower	CCT1B	20		x		x	
CCT	Climate Change Tower	CCT1F1		x				
CCT	Climate Change Tower	CCT1F2		x				

**Table 8.** List of collected gas samples for the third expedition, October 2022.

SiteID	Site name	CollectionID	Depth (cm)	AC sample	Glass vial	Vacutainer	Gas bag	Notes
BY1		BY1A	40			x		
BY1		BY1A	40			x		
BY1		BY1A	40		x		x	
BY1		BY1B	20			x		
BY1		BY1B	20			x		
BY1		BY1B	20		x		x	
BY1		F7		x				
BY1		F8		x				
BY1		F9		x				
BY1		F10		x				
BY1		F11		x				
BY1		F12		x				
BY2		BY2A	20			x		water @40 cm
BY2		BY2B	20			x		water @40 cm
BY2		BY2A	20		x		x	water @40 cm
BY2		F13		x				
BY2		F14		x				
BY2		F15		x				
BY2		F16		x				
BY3A		BY3AA	40			x		
BY3A		BY3AA	40			x		
BY3A		BY3AA1	40			x		
BY3A		BY3AA2	40			x		
BY3A		BY3AA	40		x		x	
BY3A		BY3AB	20			x		
BY3A		BY3AB	20			x		
BY3A		BY3AB	20		x		x	
BY3A		F17		x				
BY3A		F18		x				
BY3A		F19		x				
BY3A		F20		x				
BY4		BY4A1	40			x		
BY4		BY4A2	40			x		
BY4		BY4A	40		x		x	
BY4		BY4B1	20			x		
BY4		BY4B2	20			x		
BY4		BY4B	20		x		x	
BY4		F21		x				
BY4		F22		x				
BY4		F23		x				
BY4		F24		x				
BY4		F25		x				
BY4		F26		x				
BY5		BY5A1	40			x		
BY5		BY5A2	40			x		
BY5		BY5A	40		x		x	
BY5		BY5B1	20			x		
BY5		BY5B2	20			x		
BY5		BY5B	20		x		x	
CCT		CCT1A	40			x		
CCT		CCT1A	40		x		x	
CCT		CCT1B	20			x		
CCT		CCT1B	20		x		x	
CCT		F1		x				
CCT		F2		x				
CCT		F3		x				
CCT		F4		x				
CCT		F5		x				
CCT		F6		x				
ML		ML1A1	40			x		
ML		ML1A2	40			x		
ML		ML1A	40		x		x	
ML		ML1B1	20			x		
ML		ML1B2	20			x		
ML		ML1B	20		x		x	
ML		FML1		x				
ML		FML2		x				
ML		FML3		x				
ML		FML4		x				
ML		FML5		x				
ML		FML6		x				
SUN2		SUN2A	40			x		
SUN2		SUN2A	40				x	
SUN2		SUN2B	20			x		
SUN2		F1SUN		x				
SUN2		F2SUN		x				
SUN2		F3SUN		x				
SUN2		F4SUN		x				

To characterize the isotopic composition of the carbon of CO _2_ released by the permafrost surface, the gas accumulating in the accumulation chamber was sampled using two pre-evacuated vacutainers, one taken at the begin of the measurement and the other after some time, i.e., at higher CO _2_ concentration. The time lapse between the two samples depends on the CO _2_ flux, in order to ensure a significant difference in CO _2_ concentration between the two samples ( Chiodini *et al.*, 2008).

### Sites and samples log

Details on the sampling locations are given in Table 1 and the underlying data ( Montemagno *et al.*, 2024). The complete list of boreholes, pit and recovered cores and gas samples retrieved on the different expeditions are reported in Table 3, Table 4, Table 5 and Table 6. Soil in the selected areas consisted mainly of silty-clay with interspersed stones of various nature. Soil density was previously reported in this area with values of 1.70×10 ^3^ kg m ^-3^ and porosities varying from 0.36 to 0.5 ( Roth & Boike, 2001).

### Site 1 - Bayelva 1 (BY1)

The Bayelva 1 site (78.921000 °N, 11.832895 °E) is located in close proximity to the German Alfred Wegener Institute for Polar and Marine Research and the French Polar Institute Paul-Émile Victor (AWIPEV) Bayelva Permafrost monitoring site ( Roth & Boike, 2001). The location is on the top of a moraine hill on the right bank of the Bayelva river.

The first sampling was performed during the winter season on February 24 ^th^ 2022. At the time of this sampling, the site was covered by 60–70 cm of snow containing a few frozen layers on the top half of the snow cover. A snow pit of 2×2 m was dug to access the soil surface. The surface CO _2_ and CH _4_ fluxes were measured as described in the field protocols on the undisturbed surface of the permafrost. Additional measurements were made after removing the musk cover. Gas samples from the accumulation chamber were sampled at one site inside the pit. On February 24 ^th^ 2022 we were unable to drill due to a malfunction of the portable core drill. On February 25 ^th^ 2022 we returned to the site and successfully completed the sampling. The snow pit was cleaned up before starting drilling operations. The obtained cores were mainly composed of fine-grained particles (silt and small rock debris). A total of 3 boreholes were drilled ( Table 3), with recoveries ranging from 20 % to 79 %. Soil gasses were sampled from borehole *a*, at the bottom, to assess the composition and isotopic signature of CO _2_. On March 5 ^th^ 2022 surface CO _2_ and CH _4_ flux measurements were performed over the snow at several points in the area of the snow pit.

**Figure 7.**
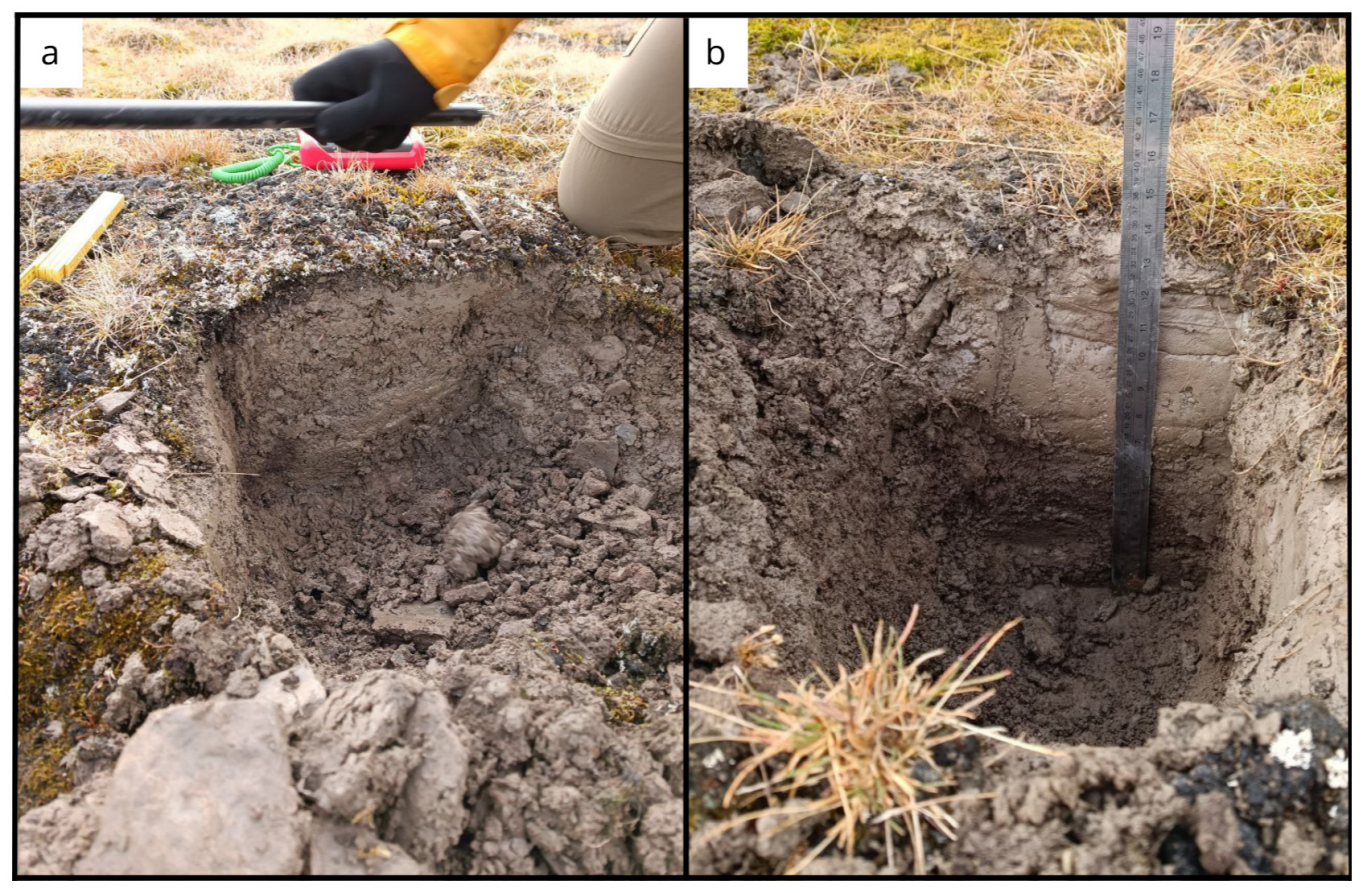
Site BY1 ( **a**) in June, after digging 30 cm of soil. In ( **b**) September we reach 35 cm of depth. Obviously without snow it was an easier sampling. Photographs taken by a member of the second expedition FM for this publication.

On the 14 ^th^ of June 2022, we started performing the second sampling. Due to the warmer temperatures, the absence of snow allowed us to choose the best spot near the site sampled in February. We made a soil pit of 40×45 cm ( Figure 7a) with a depth of 30 cm using a shovel; the surface soil temperature was 7.8 °C ( Table 1). The site was characterized by clayey soil. We took samples of soil at 3 different depths ( Table 4). In this season the soil was saturated with meltwater, and liquid water was present in the soil pit. For this reason, we collected, together with the usual samples, water samples. We took three sterivex filters (0.22 μm) after having filtered 360 mL of water for each one of them. We also collected two 50 mL falcons of filtered water and a 10 mL vial. Soil gas samples were collected using the stainless steel probe inserted into the soil next to the pit. Soil CO _2_ and CH _4_ flux measurements were performed at several points in the area of the pit. At several locations around the pit gas samples from the accumulation chamber were also collected. During the sampling of this site on the second expedition, we also collected approximately 1 kg of sediment near our soil pit to conduct a microcosm experiment to track the release of greenhouse gasses by the microbial community.

The third and last sampling was performed on the 30 ^th^ of September 2022. At this time, there was no liquid water in the area and the tundra was mostly dry. We collected soil samples at three depths ( Table 5). The soil was still thawed, and we proceeded as the second sampling, avoiding the use of the drill. Soil gas samples were collected using the stainless steel probe inserted into the soil next to the sediment sampling point. Soil CO _2_ and CH _4_ flux measurements were measured at several locations in the area of the sampling point. At several locations, gas samples from the accumulation chamber were also collected.

### Site 2 - Bayelva 2 (BY2)

**Figure 8.**
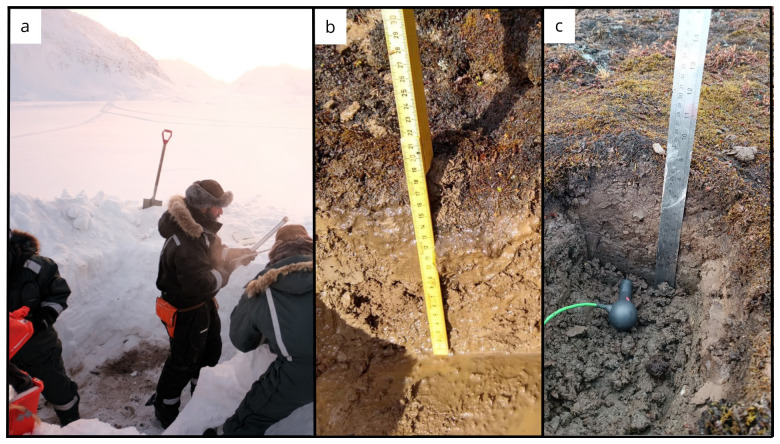
( **a**) Midday at the site Bayelva BY2 before starting the gas flux measurements during the first expedition. ( **b**) In the second expedition we observed that the area of our site was covered with rocks, and the terrain was saturated with water. ( **c**) In September, there was significantly less water and we were able to excavate to a greater depth. Photographs taken by members of the first expedition JP and FM for this publication.

The Bayelva 2 site (78.92315 °N, 11.83403 °E) is located 232 m northward from Bayelva 1 site, and it is the second site of a transect that follows the Bayelva river and goes from the main river watershed to the sea. The site is located on a flat area on the right bank of the Bayelva river and is surrounded by two main moraine hills (Kolhaugen and Leirhaugen) ( Figure 8a). At the time of sampling, the site was covered by 80–90 cm of snow containing a few frozen layers on the top half of the snow cover. On February 27 ^th^ 2022 a snow pit of 2×2 m was dug, and a total of 3 boreholes were drilled ( Table 3). The obtained cores were mainly composed of fine-grained particles (silt and small rock debris), with recoveries ranging from 71 % to 145 %. Soil gasses were sampled from boreholes *a* and *b* inserting the probe up to the hole bottom. The surface CO _2_ and CH _4_ fluxes were measured as described before inside the pit and at one location gas samples from the accumulation chamber were sampled. CO _2_ and CH _4_ fluxes measurements from the snow surface were performed on March 5 ^th^ 2022.

We sampled the BY2 site for the second time on June 14 ^th ^2022 ( Table 2). We made a 35×40 cm pit that had a surface temperature of 5.8 °C and was 25 cm in depth ( Figure 8b). We collected samples at three depths ( Table 4). During this time of the year, we did not find snow and the sediment we collected was gravelly.

On 30 ^th^ September 2022 during the third expedition we took the same number of samples in this site ( Figure 8c, Table 3 and Table 5). The surface temperature was 4.9 °C.

In both the second and third expedition soil gas samples were collected using the stainless steel probe inserted into the soil in the proximity to the soil pit, and soil CO _2_ and CH _4_ flux measurements were performed in the area of the pit and gas samples from the accumulation chamber were collected at several flux measuring points ( Table 6, Table 7 and Table 8)

### Site 3 - Bayelva 3 (BY3)

**Figure 9.**
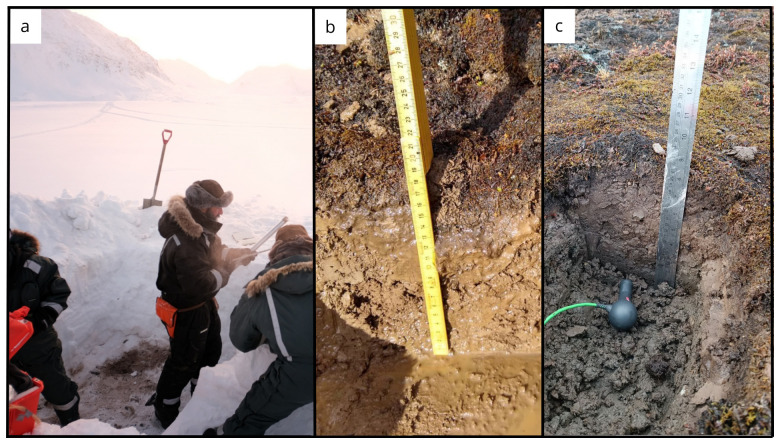
( **a**) Core extrusion operations at site Bayelva BY3 in February. ( **b**) In June, the site BY3 was located close to the river banks and, as a result, the soil was filled with sand and water. To address this issue, we relocated BY3 (BY3A) to sample the soil. In September ( **c**) samples were collected from both sites (BY3 and BY3A). Photographs taken by members of the first expedition JP and FM for this publication.

The Bayelva 3 site (78.92743 °N, 11.82908 °E) is located 492 m northward from the previous site, Bayelva 2, following the transect direction towards the sea. The site is located in the floodplain area of the Bayelva river ( Figure 9a). At the time of sampling, the site was covered by 100–105 cm of snow containing a few frozen layers on the top half of the snow cover. A 2×2 m snow pit was dug on February 27 ^th^ 2022. Three boreholes were drilled ( Table 2), with recoveries ranging from 46 % to 175 %. The obtained cores were mainly composed of fine-grained particles (silt and small rock debris).

Soil gasses were sampled from the borehole *a* bottom. The surface CO _2_ and CH _4_ fluxes were measured as described before leaving the permafrost undisturbed and at one location gas samples from the accumulation chamber were collected. The surface CO _2_ and CH _4_ fluxes measurements from the snow surface were performed on March 5 ^th^ 2022.

We sampled this site for the second time on 14 ^th^ June 2022. Once on site, we observed that the melt river flowed very close to the original BY3 location and the soil was rich in water. We sampled a single depth at this location characterized by silt and sand and relocated for sampling on the river embankment where we made a second pit BY3A (78.926336 °N, 11.826033 °E) ( Figure 9b). From this 40×45 cm pit we took samples from three depths. We also sampled river waters for microbiological and geochemical analyses. Near the BY3A site, we also collected approximately 1 kg of sediment during the second expedition, as we did near BY1 during the same expedition.

On 30 ^th^ September 2022 we repeated the sampling for the third time ( Figure 9c). We took soil samples at one depth in BY3 and at three depths in BY3A, and we also collected fluid samples from the river. We also took three sterivex filters (0.22 μm) after having filtered 360 mL of water for each one of them.

In June and September 2022 soil gas sampling, CO _2_ flux and CH _4_ flux measurements were performed at BY3A and its surrounding. Soil gas samples were collected using the stainless steel probe inserted into the soil. Gas samples from the accumulation chamber were collected at several flux measuring points.

### Site 4 - Bayelva 4 (BY4)

The Bayelva 4 site (78.93435 °N, 11.84205 °E) is located 825 m NNE from the previous site, Bayelva 3, and it is located on the riverbank of the Bayelva, close to the river’s delta. This site was sampled on February 28 ^th^ 2022. At the time of sampling, the site was covered by 19 cm of snow, without any frozen layer, and a 2×2 m snow pit was dug. Before reaching this site, two other sites were inspected, but rock and ice layers did not allow for drilling. Moving closer to the riverbank allowed us to find a better spot for the drilling. The surface CO _2_ and CH _4_ fluxes were measured as described before leaving the permafrost undisturbed. Three boreholes were drilled ( Table 3), with recoveries ranging from 54 % to 87 %. Soil gasses were sampled from borehole *a* to assess both composition and isotopic signature. The obtained cores were mainly composed of fine grained particles (silt and small rock debris). The surface CO _2_ and CH _4_ fluxes measurements from the snow surface were performed on March 5 ^th^ 2022.

**Figure 10.**
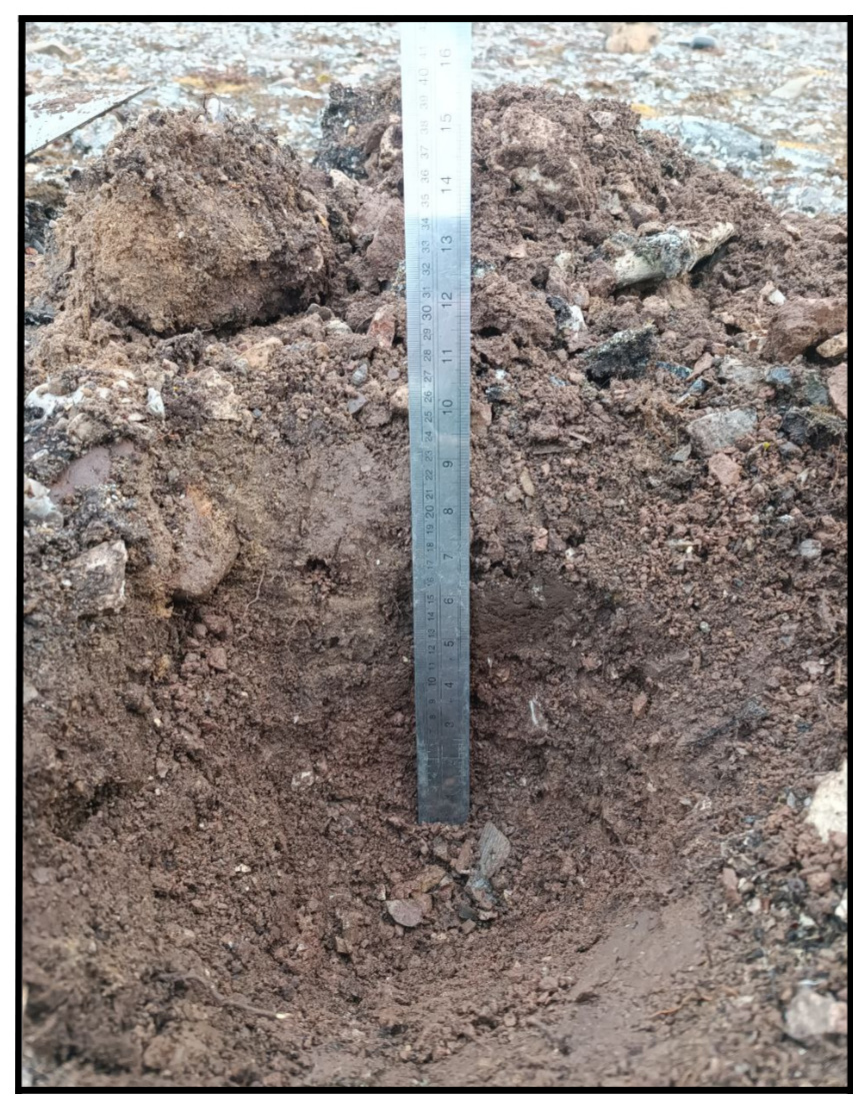
The BY4 site during the last expedition, 27 cm of depth was reached ( Table 4). Photograph taken by members of the third expedition FM for this publication.

The second sampling of this site, during the summer campaign, was programmed on the 16 ^th^ June 2022. The presence of polar bears nearby the site made us postpone the sampling up to the 19 ^th^ June 2022. The surface soil temperature was 9.2 °C and we took three soil samples from the 40×40 cm soil pit. During the third expedition, we collected three soil samples from different depths on October 3 ^th^ 2022 ( Figure 10). River water at this site was sampled during the September expedition for microbiological and geochemical analyses. River water temperature was 1.7 °C. We took three sterivex filters (0.22 μm) after having filtered 180 mL of water for each one of them.

In June and October 2022 campaigns soil gas samples were collected using the stainless steel probe inserted into the soil, CO _2_ flux and CH _4_ flux measurements were performed at several points and gas samples from the accumulation chamber were collected at several flux measuring points.

### Site 5 - Bayelva 5 (BY5)

The Bayelva 5 site (78.93613 °N, 11.85982 °E) is located 434 m ENE from the previous site, Bayelva 4, and it is located in the Bayelva river’s delta. At the time of sampling, the site was covered by 48.5 cm of snow and 20 cm of ice. The site was sampled on February 28 ^th^ 2022, and a 1×1 m snow pit was dug. The surface CO _2_ and CH _4_ fluxes were measured as described before, leaving the permafrost undisturbed. In this site, because of the large amount of rocks and ice, we were able to drill a single borehole ( Table 3), with a recovery of 111 %. Soil gasses were sampled from the borehole to assess both composition and isotopic signature. The obtained core was mainly composed of fine grained particles (silt and small rock debris). The snow surface CO _2_ and CH _4_ flux measurements were repeated on March 5 ^th^ 2022.

In the summer BY5 was sampled on June 19 ^th^ 2022 in a location closest to the sea and in close proximity with the winter site. We collected soil samples at two depths and sampled water in the proximity of the soil pit. Soil surface temperature was 7.7 °C.

**Figure 11.**
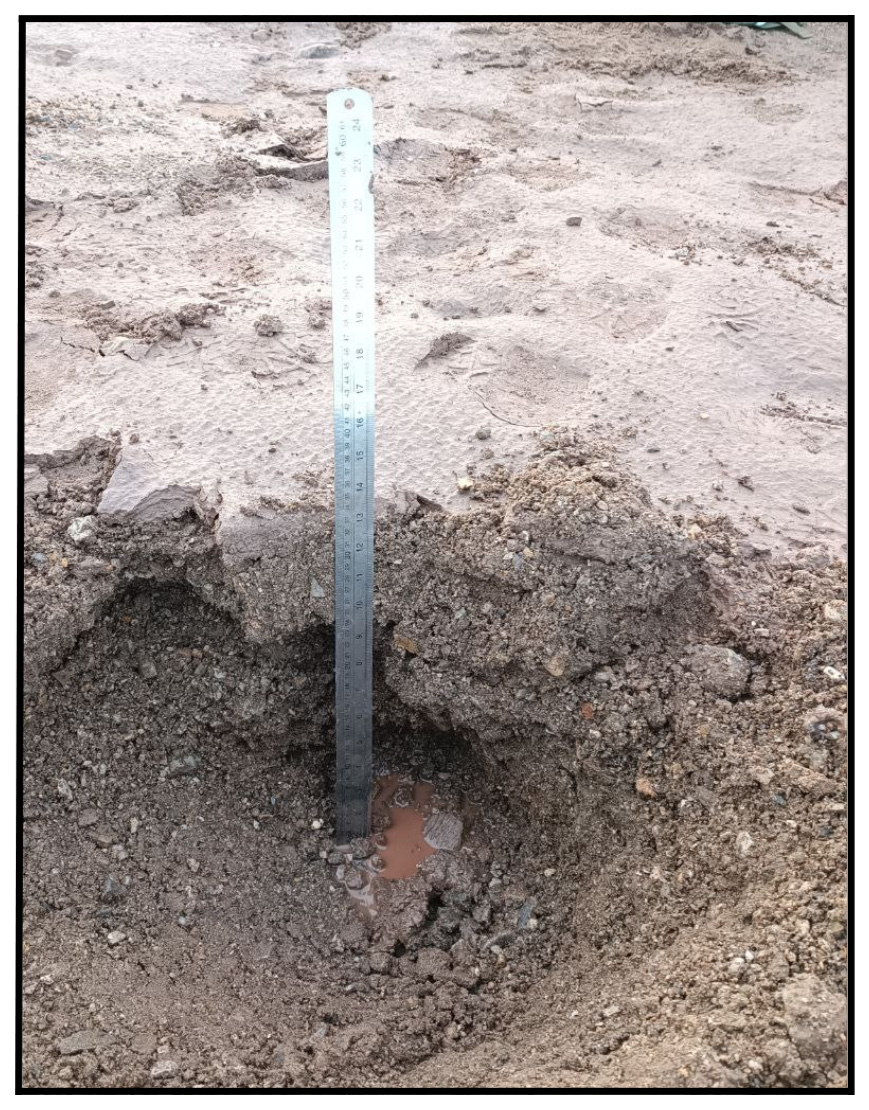
The BY5 site during the September expedition ( Table 4). Photograph taken by members of the third expedition FM for this publication.

On the third expedition in September 2022 we sampled four different depths in the BY5 site and we also sampled the water river that had a temperature of 1.7 °C. The sampled location was approximately 30 m away from the previous location due to a change in the river course compared to June 2022 ( Figure 11).

In June and September 2022 sampling campaigns soil gas samples were collected using the stainless steel probe inserted into the soil, CO _2_ flux and CH _4_ flux measurements were performed at several points and gas samples from the accumulation chamber were collected at several flux measuring points.

### Site 6 - Midtre Lovénbreen 1 (ML1)

The Midtre Lovénbreen 1 site (78.90833 °N, 12.08157 °E) is located in a plain in front of the glacier Midtre Lovénbreen between the two main melt channels. This site was chosen as more representative of tundra soils and devoid of moraine deposits, unlike previous sites where rocks were often found. The ML1 site was sampled two times in the first expedition, the first on March 1 ^st^ 2022 and the second on March 3 ^rd^ 2022. On the first day of sampling a 2×2 m snow pit was dug and three boreholes were drilled ( Table 3), with recoveries ranging from 130 % to 157 %. The obtained cores were mainly composed of fine grained particles (silt and small rock debris). Soil gasses were sampled from borehole *a* to assess both composition and isotopic signature. During the first day, radio silence imposed for the Ny-Ålesund area due to ongoing radiometric measurements at the local station did not allow for the use of the bluetooth device for the measurement of CO _2_, CH _4_ and H _2_S fluxes. On the second sampling, two 1×1 m snow pits were dug to measure the surface CO _2_, CH _4_ and H _2_S fluxes leaving the permafrost undisturbed.

**Figure 12.**
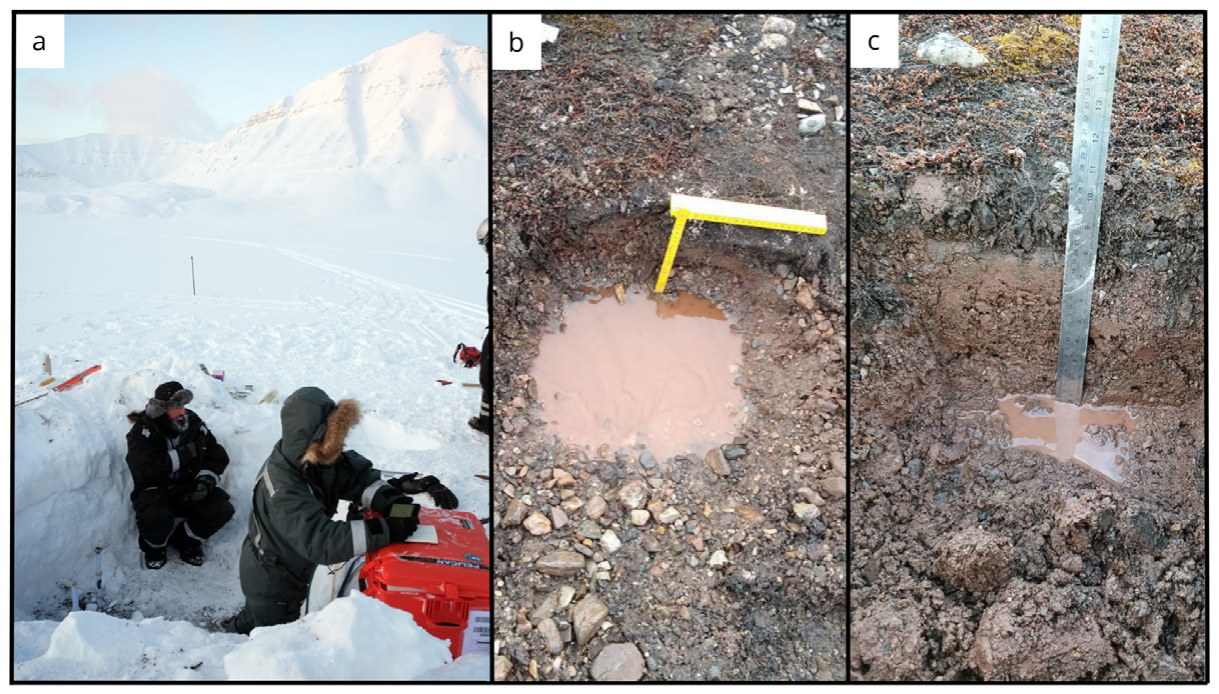
( **a**) In June, the soil exhibited a high moisture content, as evidenced by the imagery from site ML1. ( **b**) However, by September, the water content had significantly decreased. ( **c**) During our most recent expedition, we were able to locate the boreholes created using the drilling equipment during the winter season. Flare gun used for scale. Photographs taken by a member of the second and third expedition FM for this publication.

During the second expedition in June we sampled soil at four different depths and sampled water from a pool near our site ( Figure 12a). The soil surface temperature was 8 °C.

Water was absent during the third expedition and soil samples were collected at five different depths ( Figure 12b). The soil surface temperature was 4.3 °C.

In June and September 2022 sampling campaigns soil gas samples were collected using the stainless steel probe inserted into the soil, CO _2_ flux and CH _4_ flux measurements were performed at several points and gas samples from the accumulation chamber were collected at several flux measuring points.

### Site 7 - Climate Change Tower (CCT)

**Figure 13.**
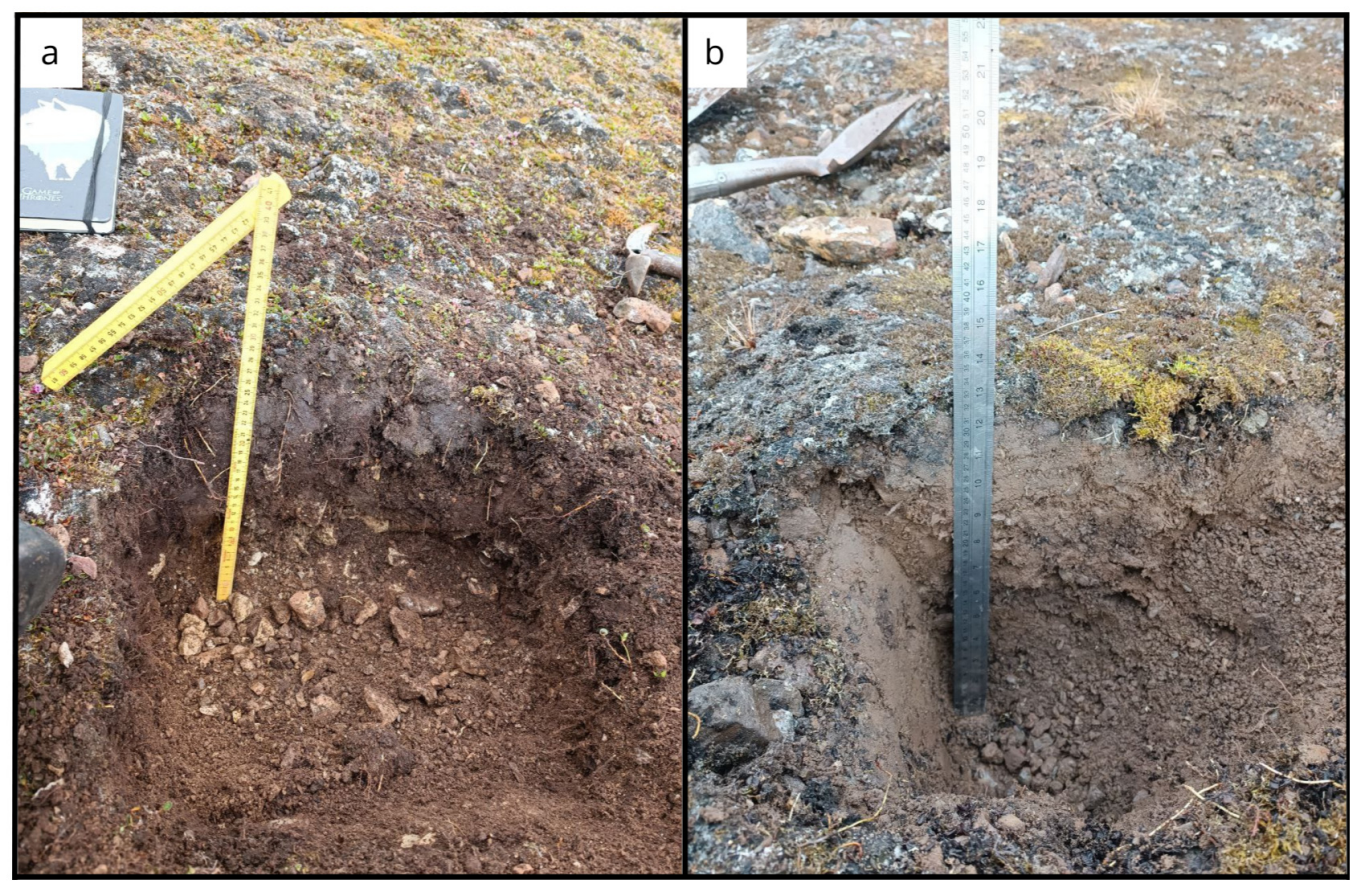
Site CCT near the Climate Change Tower in ( **a**) October and ( **b**) June. Photographs taken by a member of the second and third expedition FM for this publication.

The CCT site (78.921367 °N 11.865867 °E) is in close proximity to the Climate Change Tower (CCT). The location is on the top of a moraine hill, overlooking the Bayelva river valley and the Bayelva permafrost observatory. This site was added during the June expedition due to the presence of polar bears near Bayelva sites BY4 and BY5. In the first sampling activity of this site, during the June expedition, we made a hole of 35 cm depth ( Figure 13a). We took samples from four different depths. In the third expedition instead, we reached 33 cm of depth ( Figure 13b) and collected samples at four different depths.

## Conclusion

The three expeditions collected coupled gas geochemistry, soil geochemistry and microbiological samples from 7 sites representative of the different conditions found in the area around Ny-Ålesund, Svalbard. The data obtained will present a clear picture of the coupling between microbiological and gas geochemistry during the seasonal melting cycle of the active layer. These data, coupled with the results of the incubation experiments carried out during the second expedition, represent a significant opportunity to characterize in detail the geo-microbiological response to active layer thaw in the high arctic.

## Data Availability

No data have been produced for this study. The expedition site and metadata have been uploaded to the CoEvolve Database ( www.coevolvedb.org) and are available through the GitHub repository https://github.com/giovannellilab/PRA_MeltingICE and made available with Zenodo: giovannellilab/PRA_MeltingICE: Data behind the PRA expedition report, under DOI https://doi.org/10.5281/zenodo.10969986 ( Montemagno *et al*., 2024). This project contains the following underlying data: Giovannellilab-PRA_MeltingICE-61b6c16 PRA_MeltingICE_table1.csv PRA_MeltingICE_table2.csv PRA_MeltingICE_table3.csv PRA_MeltingICE_table4.csv PRA_MeltingICE_table5.csv PRA_MeltingICE_table6.csv PRA_MeltingICE_table7.csv PRA_MeltingICE_table8.csv README.md Data is available under the terms of the Creative Commons Attribution 4.0 International
