# Supplementary material for: PRA Melting-ICE Project: Svalbard 2022 Expeditions Report
Source: Open Res Eur. 2024 Dec 4;4:257. [Version 1] doi: 10.12688/openreseurope.17772.1 (PMC11791401; doi:10.12688/openreseurope.17772.1)
Supplement: Peer review history

# Reviewer report: Reviewer response for version 1

**DOI:** 10.21956/openreseurope.19214.r49886

**Published:** 2025-02-28

**Authors:** Edgardo A Hernández

**Affiliations:** 1 Instituto Antártico Argentino, Buenos Aires, Argentina

The report of the PRA Melting-ICE project, carried out in the Ny-Ålesund area of ​​the Svalbard archipelago, describes the three scientific expeditions in winter, summer and autumn 2022.

 The text describes in minute detail the soil sampling at different depths, and the gas emissions,and NH _4_.

 The field work required a great logistical effort due to adverse weather conditions, mainly in winter. This offers a large amount of data that will provide information on the relationship between microbial diversity, soil characteristics and gas flows and relate them to global warming in the Arctic.

 But it does not report how the soil samples are analyzed in the laboratory to study their characteristics. Nor does it explain what methodology will be used to isolate the DNA of microorganisms, to know the structure of the soil microbial community, and its spatial (in the seven sampling sites) and temporal (in the three seasons of the year) variations.

 The text is very long, which makes it difficult for readers to read the report. The tables are extensive and difficult to relate to the development of the text. Some sections require clarification to improve accessibility and scientific accuracy.

 The photos are very good, but some are very repetitive, such as those of the ruler measuring the depth of the well. It is wrong to use the same photos in Figures 8 and 9, since the titles are different. In fact, the title of Figure 8 says Bayelva BY2 and that of Figure 9 says Bayelva BY3.

 In the Conclusion they say that the data obtained, “coupled with the results of the incubation experiments carried out during the second expedition,” but they do not report these results.

 I am convinced that the processing of these valuable data will allow a detailed characterization of the geomicrobiological response to ice melting and environmental changes in the Arctic.

Are sufficient details of methods and materials provided to allow replication by others?

Partly

Is the rationale for creating the dataset(s) clearly described?

Yes

Are the datasets clearly presented in a useable and accessible format?

Partly

Are the protocols appropriate and is the work technically sound?

Yes

Reviewer Expertise:

Antarctic Microbial Ecology and Molecular Biology

I confirm that I have read this submission and believe that I have an appropriate level of expertise to confirm that it is of an acceptable scientific standard, however I have significant reservations, as outlined above.

# Reviewer report: Reviewer response for version 1

**DOI:** 10.21956/openreseurope.19214.r49884

**Published:** 2025-02-05

**Authors:** Guitao Shi

**Affiliations:** 1 East China Normal University, Shanghai, China

The PRA Melting-ICE Project report offers a comprehensive overview of the 2022 research expeditions conducted in the Ny-Ålesund area of the Svalbard archipelago. As part of the Italian Arctic Research Program, this initiative examines the microbiological and geochemical dynamics of the active layer in permafrost environments over a full seasonal cycle. The report details three expeditions winter, summer, and autumn designed to investigate microbial community responses and greenhouse gas fluxes associated with permafrost thaw. Data collected from seven distinct sites, including field observations and laboratory analyses, provide critical insights into the coupling between microbial diversity, gas fluxes, and climate impacts in the Arctic. 

 The report effectively underscores the importance of studying Arctic ecosystems, given their accelerated warming and significant contributions to global greenhouse gas emissions. Despite logistical challenges such as extreme weather and polar bear activity, the expeditions successfully obtained diverse samples and datasets that will be crucial for future analyses. While the report is detailed and highlights the vital role of permafrost thaw in climate change, certain sections require clarification and improvement to enhance accessibility and scientific precision.

 Comments to the Authors:

 1. Terminology Consistency: Terms like "Onshore Science Party" and "Support Science Party" are inconsistently used. Unifying these terms would improve clarity.

 2. Figures: Figures 8 and 9 duplicate images without clear justification. Ensure unique visuals or proper explanations for repeated usage. In addition, it is recommended to include a north arrow, scale, and longitude/latitude on the map. On P20, I cannot find a pool from Figure 12a following your description “and sampled water from a pool near our site (Figure 12a).” The caption for Figure 12 does not convey information in a clear manner, and it might be beneficial to enhance its clarity.

 3. Dataset Summarization: Summarize key findings or highlights from the datasets within the report to enhance usability for readers.

 4. Sampling Challenges: Elaborate on how challenges like equipment malfunctions and extreme weather were addressed to guide future research efforts. In addition, I don't think the wind chill temperature is an objective physical parameter.

 5. Instrumentation Rationale: Provide a brief explanation for selecting specific instruments to improve the report's technical rigor.

Are sufficient details of methods and materials provided to allow replication by others?

Yes

Is the rationale for creating the dataset(s) clearly described?

Yes

Are the datasets clearly presented in a useable and accessible format?

Partly

Are the protocols appropriate and is the work technically sound?

Yes

Reviewer Expertise:

Polar Geochemistry and Polar Environment

I confirm that I have read this submission and believe that I have an appropriate level of expertise to confirm that it is of an acceptable scientific standard.

# Reviewer report: Reviewer response for version 1

**DOI:** 10.21956/openreseurope.19214.r49882

**Published:** 2025-02-03

**Authors:** Ze Ren

**Affiliations:** 1 Nanjing Institute of Geography and Limnology, Chinese Academy of Sciences, Nanjing, China

This manuscript presents a detailed and well-organized account of the three expeditions conducted in Ny-Ålesund, Svalbard, focusing on the seasonal changes in microbial communities and greenhouse gas fluxes. The study is interesting and important given the rapid warming of the Arctic and its implications for global climate change. I only have several comments bellow:

 While the manuscript provides a thorough description of the expeditions and methodologies, it lacks data analysis or results. The authors mention that the data will be analyzed in future studies, but including preliminary findings or trends would significantly strengthen the manuscript.

 Some sections of the manuscript, particularly the descriptions of the sampling sites and procedures, are repetitive. For example, the details of the snow pits and boreholes are mentioned multiple times across different sections. Streamlining these sections would improve the flow of the manuscript.

 Fig 8 and Fig 9 are the same

Are sufficient details of methods and materials provided to allow replication by others?

Yes

Is the rationale for creating the dataset(s) clearly described?

Yes

Are the datasets clearly presented in a useable and accessible format?

Partly

Are the protocols appropriate and is the work technically sound?

Yes

Reviewer Expertise:

cryosphere, biogeochemistry, limnology

I confirm that I have read this submission and believe that I have an appropriate level of expertise to confirm that it is of an acceptable scientific standard.

# Reviewer report: Reviewer response for version 1

**DOI:** 10.21956/openreseurope.19214.r48601

**Published:** 2025-01-09

**Authors:** John Yackel

**Affiliations:** 1 University of Calgary, Calgary, Alberta, Canada

This PRA Melting-ICE Project: Svalbard 2022 Expeditions Report is well described and documented. I checked all the links in the Data and software availability section and they are all complete and working fine. While I don't believe that all authors contributed equally to this report, the statement of such says to me that the research team members served cordially and professionally towards its completion. I especially enjoyed the conciseness and readability of the report. It will serve as valuable a metadata report towards characterizing the geo-microbiological response to active layer thaw in the high Arctic with continued warming. 

 Pg 5 .... use consistent terminology wrt to *Onshore* and *Support* Science Party. Are these the same? 

 While the specific dates for sampling are *described* in each of the various Site sections (pages 10-20) by SiteID, Site name and/or CollectionID, I would have liked to see the specific sampling **dates** also listed in various Tables. I'm not sure why this wasn't or can't be done. It would make for finding the summary parameters in the tables much easier if dates from the text (to tables) could be matched. Another example of being unspecific with date/time of measurement is the caption of Figure 8. The date of this 'Midday' measurement sample. 

 Similarly, I'm not sure why dates of different photographs taken in the various Figures can't be mentioned. It is useful data. 

 Why are the same photos used in both Figures 8 and 9 but the captions are quite different? In fact, Fig 8 caption says Bayelva BY2 and Fig 9 caption says Bayelva BY3. 

 Figure 12 caption. I do not see the flare-gun used for scale in b) or c) but the different rulers are useful.

Are sufficient details of methods and materials provided to allow replication by others?

Yes

Is the rationale for creating the dataset(s) clearly described?

Yes

Are the datasets clearly presented in a useable and accessible format?

Yes

Are the protocols appropriate and is the work technically sound?

Yes

Reviewer Expertise:

microwave interactions with snow-covered seasonal sea ice

I confirm that I have read this submission and believe that I have an appropriate level of expertise to confirm that it is of an acceptable scientific standard.
